# Association between oxytocin and S100B in community‐dwelling older adults

**DOI:** 10.1002/pcn5.70130

**Published:** 2025-06-05

**Authors:** Ryuzo Orihashi, Narumi Fujino, Yoshito Mizoguchi

**Affiliations:** ^1^ Institute of Nursing Faculty of Medicine, Saga University Saga Japan; ^2^ Department of Psychiatry Faculty of Medicine, Saga University Saga Japan

**Keywords:** cognitive function, mental health, older adults, oxytocin, S100B

## Abstract

**Aim:**

The aging of the global population has made healthy aging and the extension of healthy life expectancy significant challenges for many societies. Mental health, including cognitive function, is critical to the quality of life of older adults. Oxytocin, a neuropeptide involved in social bonding and stress regulation, has been shown to exert neuroprotective effects, while S100B, a calcium‐binding protein, has been linked to neuroinflammation and neurodegenerative disorders, such as Alzheimer's disease. However, the relationship between oxytocin and S100B levels during aging remains unclear. This study investigated the association between serum oxytocin and S100B levels in community‐dwelling older adults.

**Methods:**

This survey, conducted between November 2016 and September 2017 in Kurokawa‐cho, Imari, Saga Prefecture, Japan, included community‐dwelling older adults aged ≥65 years. Blood samples were collected to measure serum oxytocin and S100B levels using an enzyme‐linked immunosorbent assay. The relationships among serum oxytocin, S100B, and cognitive function (Mini‐Mental State Examination, Frontal Assessment Battery, and Clinical Dementia Rating) were analyzed using correlation and multiple regression analyses.

**Results:**

A total of 95 participants (25 men, 70 women; mean age: 78.03 ± 5.12 years) were analyzed. Our analysis showed that serum oxytocin levels were negatively associated with serum S100B levels even after adjusting for age, sex, years of education, and body mass index. However, no significant correlations were found between these biomarkers and overall cognitive function.

**Conclusion:**

These findings suggest that the neuroprotective effects of oxytocin may influence blood S100B levels, though its direct role in cognitive function remains unclear.

## Graphical abstract

Serum oxytocin levels were negatively associated with serum S100B levels in older adults. Findings suggest oxytocin protects against neuroinflammation and neurodegeneration. Neuroprotective effects of oxytocin may influence blood S100B levels.

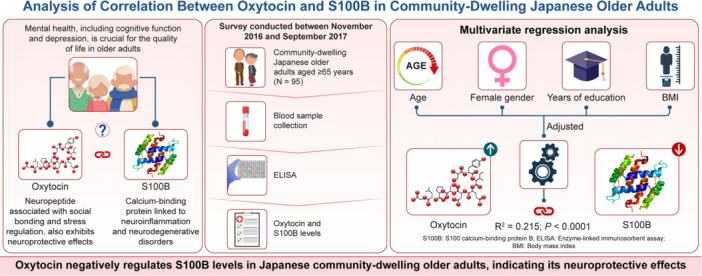

## INTRODUCTION

The global population is aging at an unprecedented rate, resulting in a steadily increasing proportion of individuals aged 65 years and older. According to the Ministry of Health, Labor, and Welfare of Japan, which governs one of the most rapidly aging societies, approximately 29% of the Japanese population is aged 65 years or older. This demographic shift presents significant challenges, particularly in promoting healthy aging and extending the period during which individuals can live independently without limitations in daily activities due to health problems, also known as healthy life expectancy. Dementia, including Alzheimer's disease (AD), is a major public health concern in Japan. According to Alzheimer's Disease International, dementia affects more than 50 million people worldwide, with a new case emerging every three seconds. Since 2004, our research group has conducted several cross‐sectional and longitudinal surveys on community‐dwelling older adults to identify reliable biomarkers for predicting the risk of future cognitive decline. These studies aimed to explore how older adults can maintain their mental health while continuing to live in familiar communities. Through these investigations, we have identified several peripheral biomarkers associated with mental health maintenance in older adults.^1^, ^2^, ^3^, ^4^, ^5^, ^6^, ^7^ Maintaining mental health and cognitive function is critical for ensuring the quality of life in older adults.

Oxytocin is a neuropeptide synthesized in the paraventricular and supraoptic nuclei of the hypothalamus. It is well known not only for its role in promoting parturition and lactation, but also for its involvement in affection and child‐rearing.^8^, ^9^, ^10^ Oxytocin is present within neuronal pathways in the brain, where it functions as both a neurotransmitter and a neuromodulator.^11^ Recent studies have further highlighted the role of oxytocin, traditionally recognized for its involvement in social bonding^12^ and stress regulation,^13^ in the mental health of aging populations.^14^ In our previous studies, serum oxytocin levels in older adults were positively associated with left hippocampal and amygdala volumes after 7 years.^7^ In addition, another study reported a positive correlation between baseline serum oxytocin levels and logical memory performance in older women after 7 years.^2^ These findings suggest that oxytocin is associated with various beneficial mental health effects in older adults, including cognitive function maintenance. Additionally, recent studies have shown that higher oxytocin levels are linked to reduced feelings of social loneliness in healthy older adults^15^ and that oxytocin counteracts the cellular aging effects of social isolation.^16^ These findings suggest that oxytocin may serve as a promising target for interventions aimed at enhancing psychological well‐being and cognitive health in older adults.

S100B is a calcium‐binding protein that serves as a classic marker of activated astrocytes. Elevated S100B levels in peripheral blood have been indicated as biomarkers for neuropsychiatric and neurological disorders.^17^, ^18^ S100B was first identified in bovine brain extracts in 1965 and is named for its solubility in 100% saturated ammonium sulfate solution.^19^ Neuroinflammation is closely associated with AD pathology. In neurodegenerative diseases such as AD, microglial and astrocytic activation has been suggested to trigger inflammatory responses in the brain, contributing to neuronal degeneration.^20^ S100B, primarily produced by astrocytes, is closely associated with astrocyte activation^21^, ^22^ and linked to the neuropathological hallmarks of AD. Further, S100B contributes to both neuroinflammation and neurotoxicity.^18^, ^23^ In addition, extracellular S100B exerts dual action, having both beneficial and detrimental functions in the brain. Specifically, at low concentrations, it is neurotrophic, whereas at higher concentrations, it is apoptotic.^24^, ^25^, ^26^, ^27^ This dual role highlights the complexity of S100B in maintaining brain function. Overexpression of human S100B has been found to exacerbate cerebral amyloidosis and gliosis in an AD mouse model.^23^ Moreover, cerebrospinal fluid S100B levels rise in the early stages of AD,^28^ and elevated serum S100B levels have been independently associated with AD.^29^ Furthermore, S100B levels positively correlate with Clinical Dementia Rating (CDR) scores and negatively correlate with Mini‐Mental State Examination (MMSE) scores in patients with AD.^30^

Despite growing interest in the neuroprotective potential of oxytocin, its relationship with S100B remains largely unexplored, particularly in the context of aging. Investigating this relationship may provide valuable insights into the mechanisms underlying mental health and cognitive function in older adults and may also inform potential clinical applications of oxytocin‐based interventions for aging‐related neuroinflammatory conditions.

In the present study, we aimed to examine the association between serum oxytocin and S100B levels in community‐dwelling individuals aged 65 years and older. Furthermore, we investigated the relationships between these serum biomarkers and cognitive function, as assessed by the MMSE, Frontal Assessment Battery (FAB), and CDR. By doing so, we hope to contribute to a deeper understanding of the role of oxytocin in age‐related neuroinflammatory processes and its potential as a biomarker and therapeutic target for promoting healthy aging and well‐being in older adults.

## MATERIALS AND METHODS

### Participant characteristics and survey procedure

This survey was conducted between November 2016 and September 2017 in Kurokawa‐cho, Imari, Saga Prefecture, Japan. The participants were community‐dwelling adults aged ≥65 years old, as reported previously.^1^, ^2^, ^3^, ^4^, ^5^, ^6^, ^7^ Kurokawa‐cho is a rural town in northwestern Saga Prefecture that is somewhat isolated from urban areas. As of 2016, the population of Kurokawa‐cho was 3137, including 935 (29.8%) people aged ≥65 years old. A power analysis was conducted to determine the necessary sample size for multiple regression analysis. Assuming a medium effect size (f² = 0.15), an alpha level of 0.05, a power of 0.80, and five predictors (oxytocin, age, sex, years of education, and body mass index [BMI]), the estimated required sample size was approximately 92 to 95 participants. This study was conducted using cross‐sectional data collected in this region between November 2016 and September 2017. Participants were primarily recruited from a list of individuals who had taken part in previous surveys that were part of a broader longitudinal study, as described in previous studies.^3^, ^4^, ^7^ Additionally, new participants who had not participated in past surveys but consented to join the study were also included. Sex ratio/distribution, current cognitive function, and activities of daily living were not considered during the participant recruitment. We asked a care manager affiliated with in‐home nursing care support offices in Kurokawa‐cho to confirm whether the selected participants could cooperate during the study.

This study was conducted in accordance with the guidelines of the Declaration of Helsinki and approved by the Ethics Committee of the Faculty of Medicine, Saga University, Japan. Written informed consent was obtained from all participants before their participation in the study.

### Collection of serum samples

Blood samples for the measurement of serum oxytocin and S100B levels were collected from participants between 1:00 p.m. and 3:00 p.m. to minimize the potential effects of diurnal fluctuations in endogenous oxytocin concentrations. Previous research has indicated that oxytocin levels exhibit a diurnal rhythm, with the lowest concentrations observed in the early morning (around 8:00 a.m.), followed by a gradual increase throughout the day, reaching peak levels in the evening (between 7:00 p.m. and 8:00 p.m.).^31^ Since human activity is generally concentrated during the daytime, excluding early morning and nighttime, we selected the afternoon period to avoid the nadir of oxytocin levels while ensuring consistency in sampling conditions. This approach helps reduce variability due to circadian fluctuations and enhances the reliability of our measurements. Samples were centrifuged at Saga University on the day of collection. Thereafter, serum was extracted from each sample, transferred to a container, and immediately stored at −80°C.

### Serum oxytocin and S100B assays

Serum samples were thawed at room temperature. All analyses were performed using duplicate serum samples. Serum oxytocin levels were measured using enzyme‐linked immunosorbent assay (ELISA), which was performed using a commercially available kit (Peninsula Laboratories International) in accordance with a previously reported non‐extraction protocol.^2^, ^5^, ^6^ The intra‐assay coefficient of variation was 13%, and the inter‐assay coefficient of variation was 9.8%. Serum S100B levels were measured using ELISA, which was performed using a commercially available kit (EMD Millipore Corporation). The intra‐assay coefficient of variation was 4% and the inter‐assay coefficient of variation was 4%. To ensure measurement reliability, quality control procedures were implemented in accordance with the manufacturer's instructions. For both oxytocin and S100B assays, blank wells and standard samples were included in each plate to establish a standard curve. Additionally, for S100B assays, quality control samples were included to monitor inter‐assay variability. The microplate reader was calibrated according to the manufacturer's recommendations to maintain measurement accuracy.

### Cognitive function assessments

All participants underwent neuropsychological assessments to evaluate various cognitive functions. The MMSE is a widely used screening tool for assessing general cognitive function.^32^ The FAB evaluates cognitive functions related to the frontal lobe.^33^ The CDR is a standardized tool used for assessing dementia severity.^34^, ^35^ These three assessments were selected because they are complementary and can collectively provide a broad overview of cognitive function while minimizing participant burden. In this community‐based study involving older adults, it was important to use tools that are brief, validated, and feasible to administer in non‐clinical settings. The MMSE provides a general overview of cognitive function, the FAB specifically assesses executive functions related to the frontal lobe, and the CDR allows for global staging of cognitive impairment. Collectively, these tools enable a multidimensional assessment of cognitive function with minimal participant burden.

### Statistical analysis

Statistical analyses were conducted using JMP statistical software (JMP 17.2.0; SAS Institute). Descriptive statistics were computed as means and standard deviations (mean ± SD) for continuous variables and as counts (*n*) and percentages (%) for categorical variables. Welch's *t*‐test and Fisher's exact test were used to compare each variable between men and women. To examine the relationship between serum biomarkers and cognitive function, Spearman's rank correlation analyses were conducted between serum oxytocin and S100B levels and cognitive function scores, including MMSE, FAB, and CDR. The relationship between serum oxytocin and S100B levels was analyzed using correlation and multiple regression analyses. In the multiple regression analysis, the dependent variable was serum S100B level, whereas the independent variables were serum oxytocin level, age, sex, years of education, and BMI. A study has reported that oxytocin levels increase with age and that women tend to have higher oxytocin levels than men.^31^ Additionally, individuals with obesity have been found to have lower oxytocin levels compared to those with normal weight.^36^ Similarly, S100B levels have been reported to be influenced by aging^37^ and to be closely correlated with BMI.^38^ Given these known associations, we included age, sex, years of education, and BMI as covariates in our analysis. These factors were selected due to their potential influence on oxytocin and S100B levels, including possible age‐related changes, sex differences in oxytocin physiology, the potential impact of education on neurodegenerative markers, and the possible role of BMI in metabolism and inflammation. Statistical significance was set at *P* < 0.05.

## RESULTS

**Figure 1:**
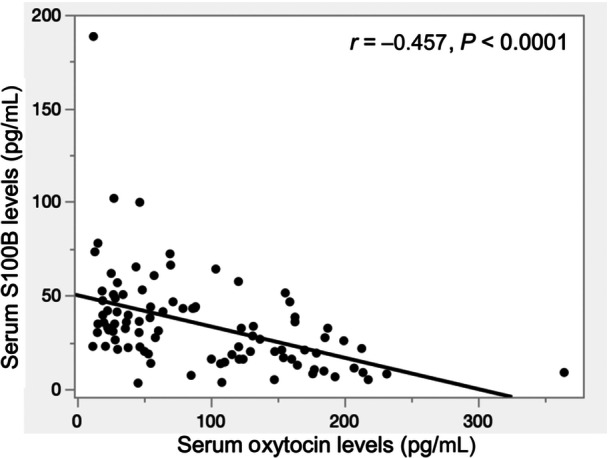
Correlation between serum oxytocin and S100B levels.

Ninety‐seven participants consented to participate in the study. Surveys were conducted once a week, with three participants evaluated in each session. Of the 97 participants, 84 had participated in previous surveys, while 13 were newly recruited. Two participants arbitrarily dropped out of the study. Finally, 95 participants (25 men and 70 women) completed the survey and were included in the cross‐sectional analysis. Given that our study included 95 participants, the sample size was considered sufficient for detecting meaningful associations. The mean ages of the men and women were 78.24 ± 3.85 years and 77.96 ± 5.52 years, respectively. There was no significant difference in serum oxytocin level between the men (81.28 ± 62.53 pg/mL) and women (98.04 ± 72.42 pg/mL) participants. Similarly, there was no significant difference in serum S100B level between men (35.00 ± 22.42 pg/mL) and women (34.32 ± 26.85 pg/mL). Furthermore, there was no significant difference in years of education, BMI, MMSE, FAB, or CDR between men and women. To further examine the magnitude of sex differences, effect sizes (Cohen's d) were calculated. Education (*d* = 0.40) showed a small‐to‐moderate effect size, while oxytocin levels (*d* = 0.24) exhibited a small effect size. Other variables, including age (*d* = 0.06), BMI (*d* = 0.03), and S100B levels (*d* = 0.03), showed negligible differences between sexes **(**Table 1). No significant correlations were found between serum oxytocin levels and MMSE, FAB, or CDR. Similarly, no significant correlations were observed between serum S100B levels and these cognitive function scores (Table 2). In addition, we conducted an exploratory analysis focusing on the MMSE recall item (0–3 points), which reflects long‐term memory. To examine the relationship with biomarkers, we dichotomized recall scores into low (0–1 points) and high (2–3 points) groups, and performed logistic regression analyses, with serum oxytocin and S100B levels examined separately as independent variables. In the unadjusted model, higher serum oxytocin levels were significantly associated with higher recall scores (odds ratio = 1.009, *P* = 0.027), while no significant association was observed for S100B. However, in the multivariable logistic regression model including age, sex, years of education, and BMI as covariates, the association between oxytocin and recall scores was attenuated and became non‐significant (odds ratio = 1.007, *P* = 0.077) (Table 3). Serum oxytocin levels negatively correlated with serum S100B levels (Figure 1). In addition, multiple regression analysis adjusted for age, sex, years of education, and BMI showed that serum oxytocin levels were negatively associated with serum S100B levels (Table 4). Moreover, to explore potential age‐related differences, we conducted a stratified analysis based on the mean age (78.03 years). The association between oxytocin and S100B remained significant in both the younger group (≤78 years: *β* = −0.547, *P* = 0.0001, *N* = 49) and the older group (≥79 years: *β* = −0.425, *P* = 0.005, *N* = 46). These findings suggest that the relationship between oxytocin and S100B persists across different age ranges, though the effect size was slightly attenuated in the older group (Table 5). Additionally, to validate the robustness of our findings, we conducted sensitivity analyses using multiple regression models, in which S100B was the dependent variable and oxytocin, age, sex, years of education, and BMI were the independent variables. First, we excluded potential outliers (values beyond ±3 SD for oxytocin and S100B, *N* = 2), and the association between oxytocin and S100B remained significant (*β* = −0.451, *P* < 0.0001). Second, we performed an alternative regression model by categorizing age into two groups (≤78 years and ≥79 years) instead of using it as a continuous variable. This approach yielded consistent results (*β* = −0.439, *P* < 0.0001). As the results were consistent across models, they are reported in the text without an additional table. These findings confirm that the relationship between oxytocin and S100B is robust and not substantially influenced by extreme values or different model specifications.

**Table 1:** Demographic characteristics of the participants.

	Overall	Men	Women		
*N*	95	25	70	*P*	Cohen's *d*
Age (years), mean ± SD	78.03 ± 5.12	78.24 ± 3.85	77.96 ± 5.52	0.781^a^	0.06
Education (years), mean ± SD	10.01 ± 1.90	10.56 ± 2.20	9.81 ± 1.76	0.135^a^	0.40
BMI (kg/m^2^), mean ± SD	23.90 ± 3.51	23.84 ± 3.04	23.93 ± 3.68	0.903^a^	0.03
Oxytocin (pg/mL), mean ± SD	93.63 ± 70.02	81.28 ± 62.53	98.04 ± 72.42	0.276^a^	0.24
S100B (pg/mL), mean ± SD	34.50 ± 25.64	35.00 ± 22.42	34.32 ± 26.85	0.902^a^	0.03
MMSE, mean ± SD	27.11 ± 2.99	26.84 ± 2.94	27.20 ± 3.02	0.605^a^	0.12
FAB, mean ± SD	13.71 ± 2.79	13.68 ± 3.08	13.71 ± 2.70	0.961^a^	0.01
CDR, *n* (%)					
0.0	86 (90.53)	22 (88.0)	64 (91.43)		
0.5	8 (8.42)	3 (12.0)	5 (7.14)		
1.0	1 (1.05)	0 (0)	1 (1.43)		
0.5 or more	9 (9.47)	3 (12.0)	6 (8.57)	0.694^b^	

Abbreviations: BMI, body mass index; CDR, Clinical Dementia Rating; FAB, Frontal Assessment Battery; MMSE, Mini‐Mental State Examination; SD, standard deviation.

^a^ Welch's *t*‐test.

^b^ Fisher's exact test.

**Table 2:** Spearman's correlation coefficients between serum biomarkers and cognitive function measures.

Variable	MMSE	FAB	CDR
Oxytocin	ρ = 0.149, *P* = 0.150	ρ = 0.153, *P* = 0.138	ρ = −0.065, *P* = 0.531
S100B	ρ = −0.036, *P* = 0.730	ρ = −0.008, *P* = 0.940	ρ = −0.069, *P* = 0.510

Abbreviations: ρ, Spearman's correlation coefficient; CDR, Clinical Dementia Rating; FAB, Frontal Assessment Battery; MMSE, Mini‐Mental State Examination.

**Table 3:** Logistic regression analysis of the association between MMSE recall score (high vs. low) and serum biomarker levels.

Independent variable	Unadjusted OR (95% CI)	*P*	Adjusted OR (95% CI)	*P*
Oxytocin	1.009 (1.001–1.016)	0.027	1.007 (0.999–1.016)	0.077
S100B	0.989 (0.972–1.006)	0.206	0.993 (0.976–1.010)	0.387

*Note*: Recall scores on the MMSE were dichotomized into two groups: low (0–1 points) and high (2–3 points). Adjusted for age, sex, years of education, and BMI. Odds ratios indicate the likelihood of higher recall scores per unit increase in biomarker levels.

Abbreviations: 95% CI, 95% confidence interval; OR, odds ratio.

**Table 4:** Multiple regression analysis with S100B as the dependent variable.

Independent variable	Model 1^a^ (*R* ^2^ = 0.214)					Model 2^b^ (*R* ^2^ = 0.215)					Model 3^c^ (*R* ^2^ = 0.215)				
	Estimate	SE	β	95% CI	*P*	Estimate	SE	β	95% CI	*P*	Estimate	SE	β	95% CI	*P*
Oxytocin	−0.164	0.035	−0.447	(−0.234, −0.094)	< 0.0001	−0.166	0.036	−0.453	(−0.237, −0.094)	< 0.0001	−0.166	0.036	−0.454	(−0.238, −0.094)	< 0.0001
Age, years	0.307	0.479	0.061	(−0.644, 1.258)	0.523	0.353	0.501	0.070	(−0.642, 1.347)	0.483	0.354	0.503	0.071	(−0.646, 1.354)	0.484
Sex (women)	1.074	2.708	0.037	(−4.304, 6.452)	0.693	1.268	2.784	0.044	(−4.262, 6.798)	0.650	1.278	2.800	0.044	(−4.285, 6.842)	0.649
Education, years						0.454	1.374	0.034	(−2.277, 3.184)	0.742	0.462	1.383	0.034	(−2.286, 3.210)	0.739
BMI											−0.097	0.688	−0.013	(−1.464, 1.270)	0.888

Abbreviations: 95% CI, 95% confidence interval; β, standardized partial regression coefficient; BMI, body mass index; SE, standard error.

^a^ Model 1: Adjusted for age and sex.

^b^ Model 2: Adjusted for age, sex, and years of education.

^c^ Model 3: Adjusted for age, sex, years of education, and BMI.

**Table 5:** Stratified multiple regression analysis by age (S100B as the dependent variable), with participants aged 78 or younger as the “Younger group” and those aged 79 or older as the “Older group.”

Independent variable	Younger group (*N* = 49) (*R* ^2^ = 0.371)					Older group (*N* = 46) (*R* ^2^ = 0.187)				
	Estimate	SE	β	95% CI	*P*	Estimate	SE	β	95% CI	*P*
Oxytocin	−0.111	0.026	−0.547	(−0.164, −0.059)	0.0001	−0.227	0.076	−0.425	(−0.380, −0.074)	0.005
Sex (women)	−1.672	2.131	−0.095	(−5.967, 2.623)	0.437	4.750	5.469	0.131	(−6.295, 15.796)	0.390
Education, years	−0.988	1.221	−0.104	(−3.449, 1.473)	0.423	1.636	2.349	0.107	(−3.107, 6.379)	0.490
BMI	−0.009	0.565	−0.002	(−1.147, 1.128)	0.987	0.034	1.282	0.004	(−2.554, 2.622)	0.979

Abbreviations: 95% CI, 95% confidence interval; β, standardized partial regression coefficient; BMI, body mass index; SE, standard error.

## DISCUSSION

In the present study, we analyzed the relationship between serum oxytocin and S100B levels in people aged ≥65 years. The results showed that serum oxytocin levels were negatively correlated with S100B levels. This association remained robust, even after adjusting for potential confounding variables, including age, sex, years of education, and BMI, using multiple regression analysis, suggesting that oxytocin's influence on S100B is independent of these factors. Moreover, to further examine the potential influence of age, we conducted a stratified analysis by dividing participants based on the mean age (78.03 years). The results demonstrated that the association between oxytocin and S100B remained significant in both groups, indicating that this relationship is not substantially modified by age. However, the effect size was slightly smaller in the older group, suggesting that other age‐related factors may contribute to the variability in S100B levels. These findings suggest that oxytocin's neuroprotective effects^39^ may modulate blood S100B concentrations. Additionally, our results provide novel insights into the interaction between oxytocin and S100B in aging populations, contributing to a better understanding of physiological processes associated with aging. Furthermore, these findings may expand the potential role of oxytocin in the psychiatric care of older adults, including those with dementia. In addition, no significant associations were found between oxytocin or S100B and cognitive function scores, including MMSE, FAB, and CDR. Although an exploratory analysis revealed a significant association between higher oxytocin levels and better performance on the MMSE recall item in an unadjusted logistic regression model, this association was no longer significant after adjusting for age, sex, years of education, and BMI. This is somewhat inconsistent with previous findings that have linked S100B to cognitive impairment in AD.^29^, ^30^ Regarding oxytocin, our findings are consistent with a previous report indicating no significant difference in plasma oxytocin levels between controls and AD patients.^40^ One possible explanation is that, as cognitive function changes over time, these biomarkers may be more closely linked to future cognitive decline rather than to current cognitive status. Longitudinal studies investigating the relationship between these biomarkers and cognitive trajectories could provide deeper insights into their potential roles in neurodegeneration.

Oxytocin is a neuropeptide primarily associated with social bonding^12^ and stress regulation.^13^ Moreover, oxytocin has been shown to reduce oxidative stress and inflammation,^41^ promote bone formation and counteract osteoporosis,^42^ and enhance muscle regeneration.^43^ These findings have recently drawn attention to oxytocin's broader physiological roles, including its anti‐aging and neuroprotective effects. Conversely, S100B is a calcium‐binding protein that influences various cellular responses along the calcium‐signal‐transduction pathway.^44^ Primarily expressed in astrocytes, S100B is closely linked to the neuropathological hallmarks of AD and is implicated in neuroinflammation and neurotoxicity.^18^, ^23^ Interestingly, extracellular S100B can exert either trophic or toxic effects, depending on its concentration. Specifically, S100B exhibits neurotrophic properties at picomolar and nanomolar levels but becomes apoptotic at the micromolar level.^24^, ^25^, ^26^, ^27^ Thus, it has been reported that S100B has a Janus face, with both beneficial and detrimental functions in the brain. Regarding the relationship between S100B and AD, a study conducted on 100 patients with AD and 100 age‐ and sex‐matched healthy controls demonstrated that serum S100B protein levels were elevated in the AD group compared to the control group and that elevated serum S100B levels and AD were independently associated.^29^ Moreover, according to a systematic review and meta‐analysis on astrocyte biomarkers in AD, blood S100B levels are significantly increased in patients with AD.^45^ Therefore, S100B in biological fluids is considered a reliable biomarker of AD.

The negative association observed between oxytocin and S100B levels in this study may indicate a regulatory mechanism through which oxytocin exerts protective effects against neuroinflammation and neurodegeneration. As mentioned previously, S100B serves as both a marker and potential mediator of AD pathology.^29^, ^45^ Additionally, elevated S100B levels have been associated with cognitive decline, a hallmark of AD.^29^ The negative association between oxytocin and S100B levels observed in the present study suggests that oxytocin may act as a mitigating factor to counterbalance S100B‐mediated neurotoxicity and inflammation. The detailed mechanisms underlying the interaction between oxytocin and S100B in the brain remain unclear. Furthermore, the extent to which peripheral oxytocin levels reflect central oxytocin concentrations remains a topic of debate. While a positive correlation has been reported, other studies have found no significant association between peripheral and central oxytocin levels.^46^, ^47^ However, our findings suggest potential clinical applications of oxytocin‐based interventions for aging‐related neuroinflammatory conditions. The use of oxytocin as a therapeutic agent, administered intranasally or through other delivery methods, could be explored as a strategy to mitigate S100B overexpression in aging populations and individuals at risk for neurodegenerative diseases. Moreover, serum oxytocin levels could serve as a biomarker for identifying individuals with heightened neuroinflammatory states, potentially enabling early interventions.

Given the observed association between serum oxytocin and S100B, oxytocin measurement may have potential as a screening tool for neurodegenerative conditions. However, its clinical applicability remains uncertain, as there is currently no direct evidence supporting its widespread use in diagnosing or monitoring dementia or other neurodegenerative diseases. Additionally, no studies have specifically examined the combination of oxytocin and S100B in older adults. Further research is necessary to determine whether oxytocin levels, in combination with other biomarkers such as S100B, can enhance the early detection of cognitive decline. Large‐scale longitudinal studies are particularly needed to validate its utility in clinical practice.

One of the major challenges in implementing oxytocin measurement in clinical settings is the lack of standardized and reliable measurement techniques. Serum oxytocin measurement is primarily conducted for research purposes, and the standardization of measurement techniques and improvement of reliability are challenges for its widespread use in clinical practice.^48^ While ELISA kits for oxytocin measurement are commercially available and commonly used in research, their application in general medical institutions remains limited due to cost constraints, technical expertise requirements, and the need for further standardization. Future studies should focus on improving measurement accuracy and reproducibility while also conducting further research that incorporates improved oxytocin measurement methods, well‐documented study subjects, and the analysis of oxytocin receptor polymorphisms to clarify the relationship between oxytocin and neurodegenerative diseases, such as AD.^49^

Oxytocin has been suggested to play a neuroprotective role.^39^ Given its association with S100B, which is involved in neuroinflammatory responses, oxytocin‐based therapeutic strategies could be explored for neurodegenerative diseases. Intranasal oxytocin has shown promise in improving neuropsychiatric symptoms in frontotemporal dementia, particularly in reducing apathy and enhancing expressions of empathy, thereby improving patient–caregiver interactions.^50^ Moreover, high‐dose intranasal oxytocin (72 IU) has been reported to yield the greatest improvement in neuropsychiatric symptoms in patients with frontotemporal dementia.^51^ While these findings suggest potential therapeutic applications, it remains unclear whether modulating the oxytocin–S100B pathway could provide similar benefits in other neurodegenerative disorders, such as AD. Further research is needed to clarify its broader therapeutic potential.

In addition to the technical and clinical challenges, the cost‐effectiveness of oxytocin measurement in routine medical practice remains uncertain. While ELISA‐based assays for oxytocin are available, their cost and feasibility in large‐scale screening programs must be assessed. The cost of oxytocin measurement varies depending on the assay used, laboratory infrastructure, and technical expertise required. In general hospitals and clinics, implementing oxytocin measurements may not be feasible due to these constraints. However, advancements in measurement technology and cost reductions could facilitate broader clinical adoption in the future. Additionally, while short‐term intranasal oxytocin administration (24–72 IU for up to 8 weeks) has been reported to be safe with mild side‐effects, its long‐term safety profile remains unclear.^52^ Further investigations into the cost‐effectiveness and safety of oxytocin‐based interventions are needed before clinical implementation.

As previously discussed, several practical considerations need to be addressed when implementing oxytocin as a therapeutic intervention. These include determining the optimal dosage and method of administration, as well as ensuring its safety with repeated and long‐term use. Studies on oxytocin administration have been conducted in both human and animal models.^50^, ^51^, ^53^, ^54^ However, as noted earlier, caution may be required when extrapolating findings from animal models to human populations. Therefore, well‐designed clinical trials are essential to establish safe and effective protocols for oxytocin administration in geriatric patients.

Future longitudinal studies are necessary to determine whether oxytocin levels predict changes in S100B concentrations over time and whether these changes correlate with cognitive and functional outcomes. Advanced imaging techniques and cerebrospinal fluid analysis can provide deeper insight into the central mechanisms linking oxytocin and S100B. Additionally, exploring the interaction between oxytocin signaling and other key markers of neuroinflammation or neurodegeneration, such as tau and amyloid‐beta, could help elucidate its broader role in aging and AD.

This study had several limitations. First, the effects of diurnal variations^31^ on serum oxytocin levels were controlled to some extent by collecting blood samples between 1:00 p.m. and 3:00 p.m. However, details regarding the participants’ activities^55^ and diet^56^ prior to the survey, as well as the medication use for physical diseases, were not obtained. Moreover, other risk factors for dementia, such as hearing loss, head trauma, hypertension, excessive alcohol consumption, smoking, depression, social isolation, and diabetes,^57^ were not available in our dataset. Therefore, the effects of these factors on oxytocin and S100B levels could not be determined. Additionally, we did not assess whether the participants regularly received psychotherapy or counseling interventions. The presence or absence of these interventions may affect the serum oxytocin levels. Second, although we adjusted for sex in our analyses, sex‐specific differences in oxytocin and S100B regulation warrant further investigation. A previous study reported that women tend to have higher levels of oxytocin than men.^31^ In our study, we did not conduct sex‐stratified analyses due to the limited sample size, but future research should explore potential sex‐based variations in oxytocin–S100B interactions. Understanding these differences could inform the development of personalized therapeutic interventions for neurodegenerative diseases. Third, our study population consisted exclusively of older adults from a rural community, which may limit the generalizability of our findings. Factors such as lifestyle (including physical activity and diet), social connections among older adults, and the prevalence of cognitive decline^58^, ^59^ and depression^60^ may differ between rural and nonrural regions. These contextual differences may influence oxytocin dynamics and cognitive outcomes, necessitating replication in diverse demographic settings. Fourth, we measured serum oxytocin levels using a commercially available ELISA kit, following an extraction‐free protocol. The kit can be used without extraction; however, the protocol for this assay recommends sample extraction prior to use. Oxytocin measurements using ELISA have been discussed in several studies. Extraction removes interfering substances^61^; however, discarded substances may include oxytocin bound to proteins.^62^ Extracted serum oxytocin levels have also been shown to be strongly correlated with unextracted serum oxytocin levels.^63^ Therefore, the debate regarding sample extraction before oxytocin assay is important.^64^, ^65^ Future studies should validate our findings using alternative quantification techniques, such as mass spectrometry or radioimmunoassay.

In conclusion, this study analyzed the relationship between serum oxytocin and S100B levels, as well as their association with cognitive function, in older adults living in rural communities. The results showed that serum oxytocin levels were negatively associated with serum S100B levels in individuals aged ≥65 years. These results suggest that the neuroprotective effects of oxytocin may influence blood S100B levels. To summarize the future research directions, it is necessary to evaluate whether baseline oxytocin levels predict longitudinal changes in S100B concentrations and cognitive decline. This would help determine whether these biomarkers serve as early indicators of neurodegeneration. Additionally, experimental studies investigating the molecular mechanisms linking oxytocin and S100B, particularly their associations with neuroinflammation and astrocyte function, may provide deeper insights into the observed relationship. Given the potential neuroprotective effects of oxytocin, clinical trials should be conducted to assess the impact of intranasal oxytocin on S100B levels, neuroinflammation, and cognitive outcomes. Identifying the optimal dosage and administration protocols while ensuring long‐term safety is crucial. Furthermore, considering the known sex differences in oxytocin regulation, future studies should include sex‐stratified analyses to determine whether the relationship between oxytocin and S100B differs between men and women.

## AUTHOR CONTRIBUTIONS

**Ryuzo Orihashi:** Writing—review and editing; writing—original draft; methodology; investigation; funding acquisition; formal analysis; data curation; conceptualization. **Narumi Fujino:** Writing—review and editing; supervision. **Yoshito Mizoguchi:** Writing—review and editing; supervision; project administration; investigation; data curation; conceptualization.

## CONFLICT OF INTEREST STATEMENT

The authors declare no conflicts of interest.

## ETHICS APPROVAL STATEMENT

This study was conducted in accordance with the guidelines of the Declaration of Helsinki and approved by the Ethics Committee of the Faculty of Medicine, Saga University, Japan.

## PATIENT CONSENT STATEMENT

Written informed consent was obtained from all participants before their participation in the study.

## CLINICAL TRIAL REGISTRATION

N/A.

## Data Availability

The data supporting the findings of this study are not publicly available, and their availability is restricted by the policies of our institutional ethics committee. However, the data are available upon reasonable request and with permission from the corresponding authors (Ryuzo Orihashi, Yoshito Mizoguchi).
